# Impact of a Community-University Partnership on the Quality of Life in a Local Community: A Validated Survey

**DOI:** 10.7759/cureus.72692

**Published:** 2024-10-30

**Authors:** Kiparissenia Samara, Areti-Dimitra Koulouvari, Evangelia-Georgia Kostaki, Theodoros N Sergentanis, Εvanthia Sakellari, Constantina Skanavis, Areti Lagiou

**Affiliations:** 1 Laboratory of Hygiene and Epidemiology, Department of Public and Community Health, School of Public Health, University of West Attica, Athens, GRC; 2 Department of Hygiene Epidemiology and Medical Statistics, School of Medicine, National and Kapodistrian University of Athens, Athens, GRC

**Keywords:** healthy cities, public health, quality of life, survey, validity

## Abstract

Background: The aim of the present study was to develop and validate a self-reported instrument exploring the perceived potential contribution and impact of a community-university partnership (CUP) on the local community’s quality of life.

Methods: A 13-item questionnaire was developed and administered to a convenience sample of 65 residents of the municipality of Egaleo, a metropolitan area of Athens, Greece, where the main campus of the University of West Attica is located. The questionnaire was self-administered and filled in at two different time points by the same participants. The 13 items of the questionnaire, pertaining to health promotion and wellness, research, upgrading of services, economic development, infrastructures, physical activity, cleaning services, green sector, social protection, kindergarten/nursery services, education, cultural sector, and technical works, were stated in the form of a three-point Likert scale. Factor analysis was performed, and the underlying factors’ reliability, convergent, and discriminant validity as well as the test-retest reliability were assessed.

Results: The mean age of participants was 46 years; 70.8% were females. All items of the questionnaire were retained in the factor analysis; the latter revealed the existence of two underlying factors, interpreted as “overall, comprehensive impact of the CUP” and “domain-specific contributions of the CUP.” High reliability (Cronbach’s alpha = 0.860 and 0.880 for the two factors, respectively) and convergent and discriminant validity were documented; the test-retest reliability indicated a stable tool for both factors (Spearman’s rho = 0.85 and 0.83, respectively, p < 0.001).

Conclusions: The instrument can be used in the future; surveys are expected to provide further, valuable insight on several aspects of the CUP.

## Introduction

Universities increasingly prioritize a public mission to provide high-quality learning experiences and advance excellence in research. This dynamic is at the heart of the principle that academic institutions should be integrally connected to the community and responsible for leveraging human, social, cultural, and economic capital to achieve progress and innovation. In this context, universities seek opportunities and strategies to more deeply involve external stakeholders in various aspects of their academic core missions, including teaching, research, and service provision. These partnerships support universities in their efforts to generate and disseminate new knowledge, as well as to improve the quality of life in local communities [1-8].

At the international level, the concept and practice of the community-university partnership (CUP) are more than two decades old; many studies have shown that mutually beneficial CUPs have the potential to promote applied research and disseminate results and outcomes that solve real-world problems while simultaneously enhancing the capacity of communities to improve their quality of life [8-27]. Universities have a plethora of expertise and competencies that can be applied to community settings so that local populations benefit from well-designed interventions. In pursuit of this mission, there is a growing demand for a participatory approach and community involvement for sustainable health promotion and social change [4,8,28-35].

CUPs often aim to improve the community’s quality of life and tackle inequalities. Community-based, participatory research and actions come through several processes and mechanisms. There is actually a variation in the methods used that may affect the quality of research and actions, the degree of community or university involvement, the broader applicability and comparability of results, and the opportunity to promote novel partnerships [6,7,17,33,36,37]. University-community engagement in participatory research and action is likely to improve the aim, objectives, and design aspects of applied research projects, increase participants’ recruitment, improve the applicability of research findings, and educate community members so as to ensure the long-term sustainability of outcomes [22,26,37-40].

Universities in Greece are in the process of developing CUPs with communities and stakeholders. The University of West Attica is an ecosystem of approximately 64,000 individuals, including students of all levels (56,190 undergraduate students), professors of teaching and research (n = 608), laboratory teaching staff/special technical laboratory staff (n = 131), and administrative staff (n = 345), as well as collaborators in various roles and forms of employment. It is the second-largest university in terms of student population in the metropolitan region of Attica (Athens, Greece) and one of the largest in Southeastern Europe, the Mediterranean, and the Balkans [41]. The two large campuses of the University of West Attica are located in the Municipality of Egaleo, Attica, Greece.

In light of the above, the aim of this study was to develop and validate a self-reported questionnaire aimed at measuring the perceived potential contributions and impacts of a CUP between the University of West Attica and the Municipality of Egaleo on various aspects of the local community's quality of life.

## Materials and methods

Study population and study tool

The research project consisting of the development and validation study of the CUP questionnaire took place in 2022. The fieldwork for the tool’s validation study was conducted in Egaleo, West Attica, where the main campus of the University of West Attica is located. The study was approved by the Ethics Committee of the University of West Attica (approval number: 70441/27-7-2022).

Sixty-five participants, derived from Egaleo residents, filled out, on a voluntary basis, the self-administered CUP questionnaire at two different time points in 2022; the time interval between the two questionnaire time points was 20 days [42]. That was a convenience sample consisting of consecutive residents of the area who visited the Municipality of Egaleo for the provision of administrative services, information, as well as health and welfare services; of 75 residents invited to participate in the survey, 65 agreed (participation rate: 86.7%).

The questionnaire was administered in hard copy form; it was self-administered, but a well-trained interviewer was present in person as a bystander to resolve any difficulties or questions potentially stemming from insufficient literacy of the participants; notably, however, no problems pertaining to the comprehension of the questionnaire items were noted.

The introductory part of the questionnaire included information that explained the study's purpose and objectives. It also involved informed consent statements regarding participation in the study and confidentiality of the data. Participants were also informed that the survey tool would take 10-15 minutes to be filled. Socio-demographic information (participants' gender, age, place of residence, socioeconomic status) was recorded as part of the questionnaire.

The main part of the questionnaire consisted of 13 closed-type questions about the potential impact of a CUP between the University of West Attica and the Municipality of Egaleo on different aspects and outcomes of the local community’s quality of life (Appendix A). The questionnaire was developed by the study authors at the University of West Attica, in collaboration with the municipality, through a meticulous analysis of the municipality's organizational chart and discussions with municipal officials regarding the services provided. Different aspects and outcomes included health promotion and wellness, research, capacity building, and service provision; economic development through attraction of resources, supplies, and building of infrastructure; promotion of physical activity; upgrading of cleaning services; the green sector; social protection; kindergarten/nursery services; cultural activities; and technical works.

To facilitate participants’ understanding, all items were worded positively. The items were stated in the form of a three-point Likert scale (where 1 represents “disagree,” 2 represents “neither agree nor disagree,” and 3 represents “agree”); a higher score indicated agreement with the item statement.

Statistical analysis

Descriptive Statistics

Means and standard deviations (SD) were used to describe continuous variables, and frequencies were used to describe categorical variables. Normality was tested by using the Kolmogorov-Smirnov test. The Spearman correlation coefficient was used to measure the strength of association between the variables under study. The significance level was set at 5%. Statistical analysis was performed by using Stata 14.2 (Stata Corp., College Station, USA) and IBM SPSS Statistics for Windows, Version 23 (Released 2015; IBM Corp., Armonk, New York, United States).

Factor Analysis

Factor analysis was used to evaluate the existence of underlying factors in our questionnaire pertaining to the CUP, as appropriate [43,44]. Data appropriateness for factor analysis was checked through the statistical measure of Kaiser-Meyer-Olkin (KMO) and Bartlett’s test of sphericity. The cut-off value for KMO was set at 0.50. A statistically significant Bartlett's test of sphericity indicates that sufficient correlations exist among the variables/items to proceed with factor analysis.

The principal components method using the correlation matrix and the varimax and promax rotation methods with Kaiser normalization was selected as the factor extraction method. Both orthogonal and oblique rotation were used to rotate the factors. Factors’ loadings for varimax (orthogonal) and promax (oblique) rotation were displayed in a rotated factor matrix and a pattern matrix, respectively. Factors were identified through the intercorrelations of a set of items; to define the number of factors that would be retained, we used the eigenvalue criterion (eigenvalue ≥ 1), the Scree plot, the cumulative percent of variance explained (at least 50%), and interpretability criteria. Each factor had to consist of at least three items with a factor loading (i.e., the partial correlation between the item and the factor) greater than 0.40. Items with factor loadings greater than 0.4 are considered stable and significant for interpretative purposes. The items with the highest factor loadings (absolute value) were considered as those that best defined each factor. In the case of cross-loadings, the ratio of loadings was calculated (cutoff value: 75%); a ratio of loadings greater than 75% indicates that the items cross-load too highly between factors [43-47]. Mean scores and SDs were also calculated for each factor.

Reliability, Convergent, and Discriminant Validity

To assess the reliability (i.e., the ability of the instrument to measure consistently [48]) of the CUP questionnaire, Cronbach's alpha coefficients were computed across all the items of the questionnaire and for items within each factor. The cutoff value for Cronbach’s alpha coefficient was set at 0.70 [48,49]. Both convergent validity (i.e., the degree to which two or more measures that are supposed to be assessing the same construct are actually related [50]) and discriminant validity (i.e., the extent to which a measure is distinct from other measures that are supposed to assess different constructs [50]) were assessed.

The corrected item-total correlation (correlation between the item score and the score of the factor to which the item belonged after the deletion of the item) was used to test factors’ convergent validity. Specifically, the higher the correlation, the more likely it is to obtain adequate convergent validity; therefore, the cut-off point for the corrected item-total correlation was set at 0.30. Furthermore, the discriminant validity of the factors was assessed by comparing the estimated correlation between the factors (Spearman’s correlation coefficient) with the factors’ Cronbach's alpha coefficients; a satisfactory discriminant validity was considered when the factors’ correlation was lower than their Cronbach's alpha coefficients [51].

Test-Retest Reliability

A test-retest reliability analysis was conducted over two time points to explore the overall concordance between the responses. In the context of our analysis, the responses of the 65 participants at two-time points (first time point: end of July and start of August 2022, for two weeks; second time point: 20 days later) were evaluated. The ratio of the sample size (i.e., 65) to the total number of items (i.e., 13) was 5:1. Spearman’s correlation coefficient was used to measure the strength of association between the sums of all 13 at each time point [51].

## Results

Descriptive statistics of the sample and responses

Descriptive statistics of the study sample are presented in Table 1.

**Table 1:** Descriptive statistics referring to the characteristics of the study sample The data are presented as frequencies and relative frequencies, i.e., n (%) except of numeric variables (age).

Categorical variables	n (%)
Gender	
Male	18 (27.7)
Female	46 (70.8)
Not disclosed	1 (1.5)
Marital status	
Married	31 (47.7)
Single	21 (32.3)
Divorced	10 (15.4)
Widowed	3 (4.6)
Occupational status	
Self-employed	3 (4.6)
Business owner	1 (1.5)
Full-time employee	49 (75.4)
Part-time employee	3 (4.6)
Unemployed	5 (7.7)
Retired	2 (3.1)
Other	2 (3.1)
Educational level	
Primary school	3 (4.6)
Secondary/High school	19 (29.2)
University/Technical University	43 (66.2)
Number of persons living in the household	
One	5 (7.7)
Two	10 (15.4)
Three	12 (18.5)
Four	20 (30.8)
Five	5 (7.7)
Six	2 (3.1)
Not disclosed	11 (16.9)
Numeric variables	Mean (SD)
Age (years)	46.0 (12.6)

The mean age of the sample was 46.0 years; 70.8% (n = 46) were females. The majority of participants were married (n = 31, 47.7%), full-time employees (n = 49, 75.4%), and had completed university or technical university studies (n = 43, 66.2%). The most common number of individuals living in the household was equal to four (n = 20, 30.8%).

In Table 2, the mean and SD of all 13 items of the CUP part of the questionnaire are presented. In all questions, participants mostly agreed that the university could have a beneficial effect on aspects, processes, and outcomes related to teaching, research, as well as the provision of services. Numerically, the highest score was found in Q2 (mean = 2.93), a question about CUP’s impact on the implementation of research; on the other hand, the lowest score was identified in Q13 (mean = 2.49), a question about the university’s contribution to technical works.

**Table 2:** Descriptive statistics regarding the 13 items of the questionnaire evaluating the effects of the CUP The data are presented as means and standard deviations (SD). The items were scored following the form of the three-point Likert scale (where 1 represents “disagree,” 2 represents “neither agree nor disagree,” and 3 represents “agree”); a higher score indicated agreement with the item statement. CUP: community-university partnership

Number	Item of the questionnaire	Mean	SD
	University of West Attica may contribute to (the):		
1	Health promotion and wellness of the municipality residents	2.89	0.37
2	Conducting research and utilizing the results for the benefit of the municipality residents	2.93	0.32
3	Upgrading services offered to the municipality residents	2.75	0.48
4	Economic development of the municipality though attracting and utilizing resources	2.80	0.45
5	Building and utilization of infrastructures	2.76	0.47
6	Promotion of physical activity in the municipality	2.70	0.53
7	Upgrading of the cleaning services sector in the municipality	2.51	0.66
8	Green sector in the municipality	2.63	0.58
9	Social protection sector in the municipality	2.63	0.58
10	Kindergartens/nursery services in the municipality	2.58	0.58
11	Field of education in the municipality	2.80	0.47
12	Cultural sector in the municipality	2.85	0.44
13	Technical works in the municipality	2.49	0.62

Results of the factor analysis

The results from the factor analysis are presented in Table 3. Both the KMO measure of sampling adequacy (0.738 > 0.50) and Bartlett’s test of sphericity, which indicates the suitability of the factor analysis model (x2 = 419.878; p-value < 0.001), verified that the data were suitable for factor analysis.

**Table 3:** Results from the factor analysis The “item-total” column denotes the correlation of the item with the overall score; the “corrected item-total” column denotes the correlation of the item with the overall score excluding the item; F1 stands for “factor 1”; F2 stands for "factor 2."

Number	Item	Item-total	Corrected item-total	Factor loading		Factor
	University of West Attica may contribute to (the):			F1	F2	
1	Health promotion and wellness of the municipality residents	0.513	0.450	0.932	-0.259	F1-overall, comprehensive impact
2	Conducting research and utilizing the results for the benefit of the municipality residents	0.710	0.669	0.816	0.017	F1-overall, comprehensive impact
3	Upgrading services offered to the municipality residents	0.711	0.652	0.576	0.262	F1-overall, comprehensive impact
4	Economic development of the municipality though attracting and utilizing resources	0.743	0.693	0.766	0.101	F1-overall, comprehensive impact
5	Building and utilization of infrastructures	0.780	0.734	0.710	0.147	F1-overall, comprehensive impact
6	Promotion of physical activity in the municipality	0.700	0.630	0.086	0.665	F2-Domain-specific contributions
7	Upgrading of the cleaning services sector in the municipality	0.644	0.559	0.254	0.419	F2-Domain-specific contributions
8	Green sector in the municipality	0.773	0.713	-0.098	0.869	F2-Domain-specific contributions
9	Social protection sector in the municipality	0.685	0.615	0.174	0.535	F2-Domain-specific contributions
10	Kindergartens/nursery services in the municipality	0.831	0.788	-0.085	0.904	F2-Domain-specific contributions
11	Field of education in the municipality	0.741	0.688	0.021	0.755	F2-Domain-specific contributions
12	Cultural sector in the municipality	0.666	0.610	0.028	0.712	F2-Domain-specific contributions
13	Technical works in the municipality	0.819	0.768	-0.057	0.866	F2-Domain-specific contributions

All commonalities (sum of squared factor loadings) were higher than 0.30, so there was no need to remove any items and rerun the analysis. The average communality was estimated at 0.6, which is considered acceptable; the average communality was reported due to the small sample size of our study (n = 65 < 300). Taking the above into account, all the 13 items were retained.

The eigenvalues and the Scree plot indicated the existence of two underlying factors, which together explained 59.7% (>50%) of the total variance. The first factor included five items (Q1 to Q5) that explored participants’ perspectives on the overall impact of the CUP upon the local community and was labeled as “Overall, comprehensive impact of the CUP.” The second factor comprised eight items (Q6 to Q13) related to participants’ perspectives on domain-specific beneficial effects of the CUP upon the community and was named “Domain-specific contributions of the CUP” (Table 3).

The mean (SD) item scores for factors 1 and 2 were 2.81 (0.36) and 2.64 (0.42), respectively, indicating the agreement of participants on the beneficial role of the CUP upon life in the local community.

Both rotations (varimax and promax) provided the same factor structure. Based on the pattern matrix, the loadings of the items on both factors were greater than 0.40, thus they were considered stable. Regarding cross-loadings, the ratio of loadings was calculated, and it was lower than the cutoff value of 75% in all cases. All the item-total correlations were well above 0.30 (recommended value), indicating a contribution to the total score by each one of them. Specifically, the item-total correlations of all items ranged between 0.513 and 0.831; all the corrected item-total correlation values were also above the recommended value of 0.30.

Factors’ reliability and validity measures

The overall Cronbach's alpha coefficient was 0.919. The Cronbach's alpha coefficient of factor 1: “overall, comprehensive impact of the CUP" was 0.860 and of factor 2: “domain-specific contributions of the CUP” was 0.880. The overall alpha coefficient and the factors’ alpha coefficients were high and well above the cut-off value of 0.70, suggesting high reliability.

Both factors had adequate convergent validity; the corrected item-total correlation values were over 0.30 for all items of factor 1 and factor 2. A satisfactory discriminant validity was noted since the factors’ correlation (rs = 0.61, p-value < 0.001) was lower than their Cronbach's alpha coefficients (factor 1: 0.860; factor 2: 0.880).

Test-retest reliability

The results of the test-retest reliability analysis are presented in Table 4. The correlation between the sums of all 13 items in each time point was found to be strong (rs = 0.92, p-value < 0.001), indicating a stable tool. The mean of the test and retest scores was 35.6 (SD = 4.8) and 35.9 (SD = 4.6), respectively.

**Table 4:** Results of the test-retest reliability analysis, overall and for each factor separately Data are presented as means and standard deviations (SD); p-value < 0.05 was considered significant. CUP: community-university partnership

	Time point	Mean (SD)	Spearman’s correlation coefficient	p-value
All items	1	35.6 (4.8)	0.92	<0.001
	2	35.9 (4.6)		
Factor				
Factor 1 - overall, comprehensive impact of the CUP	1	14.0 (1.8)	0.85	<0.001
	2	14.1 (1.5)		
Factor 2 - domain-specific contributions of the CUP	1	21.2 (3.3)	0.83	<0.001
	2	21.4 (3.4)		

Moreover, the test-retest reliability statistics were estimated separately for each factor. Regarding factor 1, “overall, comprehensive impact of the CUP," a strong correlation between test and retest scores was detected (rs = 0.85; p-value < 0.001). A strong correlation between test and retest scores was also found for factor 2 “domain-specific contributions of the CUP” (rs = 0.83, p-value < 0.001). These strong correlations between test and retest scores indicated a high test-retest reliability for both factors.

## Discussion

The CUP assessment tool, validated in this study, has demonstrated excellent properties and could be used in further sizable surveys, which might provide valuable insight into different aspects of CUPs and useful recommendations for evaluating the long-term impact on outcomes and the short-term contribution on processes of these partnerships in local communities.

There has been a growing body of literature on the changing role and functions of universities, being closely connected to the community for the mutually beneficial exchange and application of knowledge and resources in the context of CUPs [16,18,28,35,36]. In line with the recent advances in the scientific community, universities in Greece are in the process of endorsing the call for the expansion of their boundaries to community stakeholders.

The two factors provided an interesting complementary duality; the first factor reflected the overall, comprehensive benefits in terms of health promotion and wellness, research, capacity building, service provision, and economic development through the attraction of resources, supplies, and building of infrastructure. The overall benefits endorsed by the local residents of the community are in line with the broad effects of the CUPs in the literature [22,26,27,37-40]; indeed, the CUPs have emerged as a valuable strategic entity in the delivery of the United Nations Sustainable Development Goals [27]. Interestingly, the study participants adopted the idea that the university is linked to the economic development of the community; although universities have been traditionally viewed as the “fountains of knowledge” contributing to the economic growth of their region [52], there has been a recent debate highlighting the challenges between the abstract knowledge generated in the universities and the tangible results necessitated by the business sector for development [52]. Notably, it has been suggested that the multitude of strategies and domains included in the CUP can elicit a series of long-term “ripple effects,” with impacts occurring outside of narrower scopes of planned projects [53].

Furthermore, factor 2 expressed the multitude of section-specific actions in which the CUP is anticipated to be beneficial. This spectrum included the promotion of physical activity, upgrading of cleaning services, the green sector, social protection, kindergartens/nursery services, cultural activities, and technical works. These pluripotent actions are in line with the published literature. Davis et al. (2017) reported the enhancing role of a CUP, promoting the implementation of physical activity recommendations, increasing walkability, and local park enhancements [54]. Apart from the community overall, such actions may also support vulnerable groups, such as persons with disabilities, as shown by Ginis et al. (2012) for a CUP focusing on patients with spinal cord injuries [55]. Regarding social protection, Dunn et al. (2020) highlighted the usefulness of a CUP between university and middle school for peace-conflict resolution against youth violence in Massachusetts [3]. With respect to the global emergency of the climate crisis, CUPs are emerging as useful tools [27], further expanding the milestones of up-to-date climate change education in universities [56].

The key concept of CUP refers to a sustained collaboration for the mutually beneficial exchange and application of knowledge and resources; this research project was undertaken at the University of West Attica [41], a large public university in the municipality of Egaleo, a metropolitan area of Athens, Greece, where the main campus of the university is located. As a major university with a broad array of professional and academic disciplines, located in an urban environment, the University of West Attica has prioritized its public mission; this mission reflects a commitment to enhancing the community’s quality of life, related to its academic work that is teaching, research, as well as service provision [41].

Among the strengths of this study, the high reliability and validity statistics, including Cronbach’s alpha (0.860 and 0.880 for the two factors) and a series of indices, should be mentioned. In addition, providing community partners with a tool evaluating the potential of the CUP may yield valuable data about the expected partnerships’ multifaceted contribution and impact, enhancing efforts for development and community support. As the size, mission, and complexity of modern universities have grown, so has the need for information to describe their efforts to further support decision-making.

Despite the strengths of our study, some limitations should be declared. First, as with the development of any tool and given the challenges in measuring the wider impact and contribution of the CUPs, the generalizability of this tool should be considered with caution, as this is a study performed in the context of a South European, metropolitan urban, and densely populated area. Second, the CUP concept is a wide umbrella concept, which may include community-based research projects, service-learning activities, university-community educational agencies, shared programs, and community-based training programs; different CUP initiatives have been and will be arising worldwide [8]. Therefore, partnership-specific aspects of them might not have been covered through the 13 items integrated in our tool that were generated through the analysis of the municipality's organizational chart and discussions with municipal officials regarding the services provided. Third, the sample size was rather limited (n = 65); nevertheless, a series of aspects could be considered adequate for exploratory factor analysis in our study [43], i.e., the ratio of sample size to number of items was 5:1, the average communality was 0.6, and the items-to-factors ratio was 13:2. In addition, the fact that convenience sampling was used could also have limited the representativeness of the sample with respect to the population of the local community. Moreover, regarding the development of the questionnaire, a Delphi algorithm was not performed.

## Conclusions

This study effectively documented the validity of a tool designed to critically evaluate the effects of a CUP within a local community. The findings from this validation study provide a valuable foundation for future research, offering insights into how local communities perceive and assess the potential impacts and outcomes of such partnerships. Moreover, these results underscore the importance of conducting comparative studies across different communities and universities to deepen our understanding of the varying effects and benefits of CUPs.

## Appendices

Appendix A

The University of West Attica (UNIWA) - Egaleo CUP Questionnaire

Here are some views regarding the potential contributions of the University to the local community. Please select the answer that best describes your perceptions.

**Table 5:** The University of West Attica (UNIWA) - Egaleo CUP questionnaire CUP: community-university partnership

Number	Item			
	University of West Attica may contribute to (the):			
1	Health promotion and wellness of the municipality residents	Disagree ☐	Neither agree nor disagree ☐	Agree ☐
2	Conducting research and utilizing the results for the benefit of the municipality residents	Disagree ☐	Neither agree nor disagree ☐	Agree ☐
3	Upgrading services offered to the municipality residents	Disagree ☐	Neither agree nor disagree ☐	Agree ☐
4	Economic development of the municipality though attracting and utilizing resources	Disagree ☐	Neither agree nor disagree ☐	Agree ☐
5	Building and utilization of infrastructures	Disagree ☐	Neither agree nor disagree ☐	Agree ☐
6	Promotion of physical activity in the municipality	Disagree ☐	Neither agree nor disagree ☐	Agree ☐
7	Upgrading of the cleaning services sector in the municipality	Disagree ☐	Neither agree nor disagree ☐	Agree ☐
8	Green sector in the municipality	Disagree ☐	Neither agree nor disagree ☐	Agree ☐
9	Social protection sector in the municipality	Disagree ☐	Neither agree nor disagree ☐	Agree ☐
10	Kindergartens/nursery services in the municipality	Disagree ☐	Neither agree nor disagree ☐	Agree ☐
11	Field of education in the municipality	Disagree ☐	Neither agree nor disagree ☐	Agree ☐
12	Cultural sector in the municipality	Disagree ☐	Neither agree nor disagree ☐	Agree ☐
13	Technical works in the municipality	Disagree ☐	Neither agree nor disagree ☐	Agree ☐

## References

[1] Chilenski SM, Welsh J, Olson J, Hoffman L, Perkins DF, Feinberg ME (2018). Examining the highs and lows of the collaborative relationship between technical assistance providers and prevention implementers. Prev Sci.

[2] Dave G, Frerichs L, Jones J (2018). Conceptualizing trust in community-academic research partnerships using concept mapping approach: a multi-CTSA study. Eval Program Plann.

[3] Dunn M, Drew C, O'Brien J, Wood M, Mora E, Diener S, Perry DJ (2020). A community-Academic Partnership for school-based nonviolence education: the healthy power program. J Sch Health.

[4] Luger TM, Hamilton AB, True G (2020). Measuring community-engaged research contexts, processes, and outcomes: a mapping review. Milbank Q.

[5] Sanders Thompson VL, Ackermann N, Bauer KL, Bowen DJ, Goodman MS (2021). Strategies of community engagement in research: definitions and classifications. Transl Behav Med.

[6] Bringle RC, Clayton P, Price M (2009). Partnerships in service learning and civic engagement. Partnerships.

[7] Bryer TA, Pliscoff C, Wilt Connors A (2020). University-community partnerships in the literature. Promoting Civic Health Through University-Community Partnerships. Rethinking University-Community Policy Connections.

[8] (2023). Routledge Handbook of university-community partnerships in planning education. https://scholar.google.com/scholar?q=intitle:Routledge%20Handbook%20of%20University-Community%20Partnerships%20in%20Planning%20Education.

[9] Minkler M, Blackwell AG, Thompson M, Tamir H (2003). Community-based participatory research: implications for public health funding. Am J Public Health.

[10] Wallerstein N, Duran B (2010). Community-based participatory research contributions to intervention research: the intersection of science and practice to improve health equity. Am J Public Health.

[11] Lamus-Lemus F, Correal-Muñoz C, Hernandez-Rincon E, Serrano-Espinosa N, Jaimes-deTriviño C, Diaz-Quijano D, García-Manrique JG (2017). The pursuit of healthier communities through a community health medical education program. Educ Health (Abingdon).

[12] Lawler JM, Rosenblum KL, Muzik M, Ludtke M, Weatherston DJ, Tableman B (2017). A collaborative process for evaluating infant mental health home visiting in Michigan. Psychiatr Serv.

[13] Marsiglia FF, Dustman P, Harthun M, Coyne Ritland C, Umaña-Taylor A (2017). Community-based effectiveness trials as a means to disseminate evidence-based and culturally responsive behavioral health interventions. Health Soc Work.

[14] Thackrah RD, Hall M, Fitzgerald K, Thompson SC (2017). Up close and real: living and learning in a remote community builds students' cultural capabilities and understanding of health disparities. Int J Equity Health.

[15] Zahnd WE, Smith T, Ryherd SJ, Cleer M, Rogers V, Steward DE (2017). Implementing a nutrition and physical activity curriculum in head start through an academic-community partnership. J Sch Health.

[16] Lemacks J, Landry A, Wenzler P (2018). Formative research to identify community partnerships and foster relationships for health promotion research in South Mississippi. Public Health.

[17] Brush BL, Mentz G, Jensen M (2020). Success in long-standing community-based participatory research (CBPR) partnerships: a scoping literature review. Health Educ Behav.

[18] Farruggia SP, Solomon B, Back L, Coupet J (2020). Partnerships between universities and nonprofit transition coaching organizations to increase student success. J Community Psychol.

[19] Pennington K, Harwood E, Sick B (2020). Characterizing the community collaborations of a community-based student-run clinic. J Prim Care Community Health.

[20] Straughan AJ, Zapanta PE, Goodman JF (2020). Building community relations and promoting cancer prevention: an oral cancer screening event. Otolaryngol Head Neck Surg.

[21] Han HR, Xu A, Mendez KJW (2021). Exploring community engaged research experiences and preferences: a multi-level qualitative investigation. Res Involv Engagem.

[22] ICBOs and Allies Workgroup (2022). Understanding the impact of equitable collaborations between science institutions and community-based organizations: improving science through community-led research. Bioscience.

[23] Weerts DJ, Sandmann LR (2010). Community engagement and boundary-spanning roles at research universities. JHE.

[24] Kennedy-Rea S, Mason J, Hereford C, Whanger S (2021). Engaging community perceptions of research in rural West Virginia. Collab J Community-Based Res Pract.

[25] Sathorar H, Geduld D (2021). A critical approach to university-community partnerships: reflecting on the diverse realities. Educ Res Soc Change.

[26] Fahrenwald C, Resch K, Rameder P, Fellner M, Slepcevic-Zach P, Knapp M (2023). Taking the lead for campus-community-partnerships in Austria. Front Educ.

[27] Leal Filho W, Dibbern T, Viera Trevisan L (2023). Mapping universities-communities partnerships in the delivery of the Sustainable Development Goals. Front Environ Sci.

[28] Skizim M, Harris N, Leonardi C, Scribner R (2017). Academic-community partnership development to enhance program outcomes in underserved communities: a case study. Ethn Dis.

[29] Tung EL, Gunter KE, Bergeron NQ, Lindau ST, Chin MH, Peek ME (2018). Cross-sector collaboration in the high-poverty setting: qualitative results from a community-based diabetes intervention. Health Serv Res.

[30] Wennerstrom A, Haywood C, Wallace M (2018). Creating safe spaces: A community health worker-academic partnered approach to addressing intimate partner violence. Ethn Dis.

[31] Kaplan SA, Gourevitch MN (2020). Leveraging population health expertise to enhance community benefit. Front Public Health.

[32] Ramírez AA, Peña MK, Cardona JA, Marín SM, Londoño GC, Vargas CE (2022). Social participation in Health: a community-based participatory research approach to capacity building in two Colombian communities. Prog Community Health Partnersh.

[33] Suarez-Balcazar Y, Balcazar F, Miranda DE, Velazquez T, Arcidiacono C, Garcia-Ramirez M (2022). Promoting justice through community-based research: International case studies. Am J Community Psychol.

[34] Curwood SE, Munger F, Mitchell T, Mackeigan M, Farrar A (2011). Building effective community-university partnerships: are universities truly ready?. MJCSL.

[35] Eddy PL (2010). Special issue: partnerships and collaborations in higher education. ASHE High Educ Rep.

[36] Addison CC, Campbell Jenkins BW, Odom D, Fortenberry M, Wilson G, Young L, Antoine-LaVigne D (2015). Building collaborative health promotion partnerships: the Jackson Heart Study. Int J Environ Res Public Health.

[37] Sarrami-Foroushani P, Travaglia J, Debono D, Braithwaite J (2014). Key concepts in consumer and community engagement: a scoping meta-review. BMC Health Serv Res.

[38] Faber JS, Al-Dhahir I, Reijnders T (2021). Attitudes toward health, healthcare, and eHealth of people with a low socioeconomic status: a community-based participatory approach. Front Digit Health.

[39] Scruby LS, Canales MK, Ferguson E, Gregory D (2017). Promoting face-to-face dialogue for community engagement in a digital age. Can J Nurs Res.

[40] Dumlao R, Janke EM (2012). Using relational dialectics to address differences in community-campus partnerships. J High Educ Outreach Engagem.

[41] (2024). UNIWA Today. https://www.uniwa.gr/en/.

[42] Davey AF, Roberts MJ, Mounce L, Maramba I, Campbell JL (2016). Test-retest stability of patient experience items derived from the national GP patient survey. Springerplus.

[43] Kline P (1993). An Easy Guide to Factor Analysis. https://doi.org/10.4324/9781315788135.

[44] Child D (2006). The Essentials of Factor Analysis.

[45] Guadagnoli E, Velicer WF (1988). Relation of sample size to the stability of component patterns. Psychol Bull.

[46] Streiner DL (1994). Figuring out factors: the use and misuse of factor analysis. Can J Psychiatry.

[47] Kaiser HF (1974). An index of factorial simplicity. Psychometrika.

[48] Tavakol M, Dennick R (2011). Making sense of Cronbach's alpha. Int J Med Educ.

[49] Cronbach LJ (1951). Coefficient alpha and the internal structure of tests. Psychometrika.

[50] Campbell DT, Fiske DW (1959). Convergent and discriminant validation by the multitrait-multimethod matrix. Psychol Bull.

[51] Ware JE Jr, Gandek B (1998). Methods for testing data quality, scaling assumptions, and reliability: the IQOLA project approach. International quality of life assessment. J Clin Epidemiol.

[52] Breznitz SM (2014). The Fountain of Knowledge. https://books.google.gr/books?hl=el&lr=&id=O3zEAwAAQBAJ&oi=fnd&pg=PR9&dq=The+Fountain+of+Knowledge&ots=CCbF4_QDYc&sig=bjw64gozl-Oj5NabIOc8KH6Zt2c&redir_esc=y#v=onepage&q=The%20Fountain%20of%20Knowledge&f=false.

[53] Zimmerman EB, Creighton GC, Miles C (2019). Assessing the impacts and ripple effects of a community-university partnership: a retrospective roadmap. Mich J Community Serv Learn.

[54] Arora A, Belenzon S, Cioaca LC, Sheer L, Zhang H (2023). The effect of public. Science on corporate R&D. NBER.

[55] Davis SM, Cruz TH, Hess JM, Kozoll R, Page-Reeves J (2017). Implementing physical activity recommendations in a tri-ethnic rural community through a community-university partnership. Prog Community Health Partnersh.

[56] Ginis KA, Latimer-Cheung A, Corkum S, Ginis S, Anathasopoulos P, Arbour-Nicitopoulos K, Gainforth H (2012). A case study of a community-university multidisciplinary partnership approach to increasing physical activity participation among people with spinal cord injury. Transl Behav Med.

